# Effect of Strontium Chloride on Experimental Bladder Inflammation in Rat

**DOI:** 10.1155/2014/369292

**Published:** 2014-10-29

**Authors:** Esat Korgali, Gokce Dundar, Kubra Acikalin Coskun, Melih Akyol, Yusuf Tutar, Semih Ayan, Gokhan Gokce, Emin Yener Gultekin

**Affiliations:** ^1^Department of Urology, Medical Faculty, Cumhuriyet University, Urology Clinic, 58140 Sivas, Turkey; ^2^Department of Bioengineering, Natural Sciences and Engineering Faculty, Gaziosmanpasa University, 60250 Tokat, Turkey; ^3^Department of Dermatology, Medical Faculty, Cumhuriyet University, 58140 Sivas, Turkey; ^4^Department of Biochemistry, Pharmacy Faculty, Cumhuriyet University, 58140 Sivas, Turkey

## Abstract

*Introduction*. Strontium salts are anti-irritants for chemically induced sensory irritation. Interstitial cystitis is a painful disease without definitive therapy. The aim of the study was to determine the effect of strontium in bladder with experimental interstitial cystitis model. *Material and Methods*. Rats' bladders in control group were instilled with NaCl. Second group was instilled with *E. coli* LPS. Third group was instilled with strontium. Fourth group was initially instilled with strontium and then LPS. Fifth group was instilled with LPS initially and then strontium. Urine of rats was collected at the beginning and end of the study. *Results*. Histamine and TNF-*α* changes were statistically significant in the second group but were not significant in the third group. When we compared the histamine levels of second via fourth and fifth groups the changes were statistically not significant. When we compared the TNF-*α* levels of second via fourth and fifth groups the changes were statistically significant. *Conclusions*. In our model, strontium did not make any significant changes in histopathology or histamine levels; however, it significantly reduced the levels of TNF-*α*. Given the role of TNF-*α* in the physiopathology of interstitial cystitis, these results suggested that further studies are required to evaluate the potential use of strontium in the management of interstitial cystitis.

## 1. Introduction and Objectives

Interstitial cystitis/painful bladder syndrome (IC/PBS) was defined by the Society for Urodynamics and Female Urology as “an unpleasant sensation (pain, pressure, and discomfort) perceived to be related to the urinary bladder, associated with the lower urinary tract symptoms for more than 6 weeks duration, in the absence of infection or other identifiable causes” [1]. It is a common problem and affects 2.7%–6.5% of all women [2]. Diagnosis of IC/PBS is based on symptoms, excluding other possible causes of those symptoms.

The etiology and pathogenesis of IC/PBS are unclear. Many possible pathophysiological mechanisms have been suggested for IC/PBS, including inflammatory, neurogenic, autoimmune, vascular, or lymphatic disorders; damage to the glycosaminoglycan layer and apoptosis of urothelium; and the occurrence of toxic substances in the urine [3].

TNF-*α* also promotes apoptosis through binding to TNF receptor 1, resulting in many effects, ranging from inflammation to apoptosis [4]. The role of TNF-*α* in the development of the IC has been revealed in various studies [5, 6]. One of these studies showed that apoptosis of urothelial cells in patients with IC/PBS could result from upregulation of inflammatory signals, including TNF-*α* [5]. Another study demonstrated that TNF-*α* induces apoptosis via TNF-related apoptosis inducing ligand (TRAIL) and its receptors in IC/PBS patient's bladder tissue samples [6].

Strontium compounds are used in the treatment of osteoporosis, in palliative treatment of metastatic prostate cancer, and as anti-irritant in dermatologic diseases [7–9]. Osteoporosis studies showed that strontium compounds inhibit apoptosis of osteoblasts by reducing the expression of TNF-*α* and inflammatory cytokines [10].

We investigated the effect of intravesical SC on the release of histamine and TNF-*α* in urine in an established animal model of bladder inflammation [11] using bacterial LPS and searched whether it would be a potential agent in the treatment of IC/PBS.

## 2. Materials and Methods

This work is supported by the Scientific Research Project Fund of Cumhuriyet University under the Project number “T-499.” Strontium chloride hexahydrate (SrCl2 6H2O) was purchased from Sigma-Aldrich (255521) and its 3% (w/v) concentration was prepared with sterile water freshly. LPS from* Escherichia coli* K-235 was purchased from Sigma-Aldrich (L2143-10 mg) and its 100 *μ*g/mL concentration was prepared with sterile water in a culture medium.

**Figure 1:**
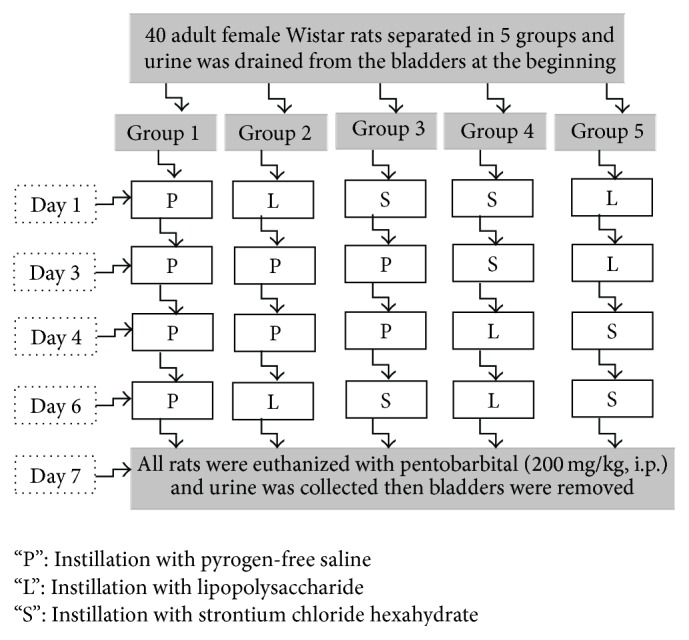
Flow chart of the study.

Adult female Wistar rats (180–230 g) were purchased from the Animal Center of the Faculty of Medicine, Cumhuriyet University, Sivas, Turkey. The Animal Research Committee of Faculty of Medicine, Cumhuriyet University, conducted the study in accordance with the guidelines for the care and use of laboratory animals. The procedure of Stein et al. was followed to instill LPS into the bladder [11]. Procedures were induced in 8 rats per group in 5 different groups. Rats were anesthetized with ketamine 200 mg/kg and xylazine 2.5 mg/kg, intraperitonealy. With its tip lubricated with glycerin a translucent 24-gauge catheter was introduced transurethrally into the bladder and advanced until the first drop of urine appeared in the hub. The urine was drained from the bladders. The bladders were instilled with 200 *μ*L of one of the following substances in 5 groups for each rat. The first group's bladders were instilled with pyrogen-free saline, the second group's bladders were instilled with LPS on days 1 and 6, and the third group's bladders were instilled with strontium on days 1 and 6. The fourth group as preventing groups' bladders were instilled initially with strontium on days 1 and 3 following LPS on days 4 and 6. The fifth group as treatment groups' bladders were instilled initially with LPS on days 1 and 3 following strontium on days 4 and 6 (Figure 1). These substances were infused at a slow rate to avoid trauma and vesicoureteral reflux on the days that were described above. Voiding of the rats was observed approximately half an hour later after the bladders were instilled. On the 7th day after the first instillation, rats were euthanized with pentobarbital (200 mg/kg, i.p.) and urine was collected and then bladders were removed.

TNF-*α* and histamine levels were detected by ELISA method in rats' urine, using Cayman (Michigan, USA) commercial kits with Thermo Multiscan HC plate reader device.

All data were expressed as mean ± SEM. Statistical comparisons between groups were performed using general linear models of analysis of variance (ANOVA) followed by the Tukey test and a *t*-test when appropriate, and *P* values of less than 0.05 were considered to be statistically significant.

## 3. Results

**Figure 2:**
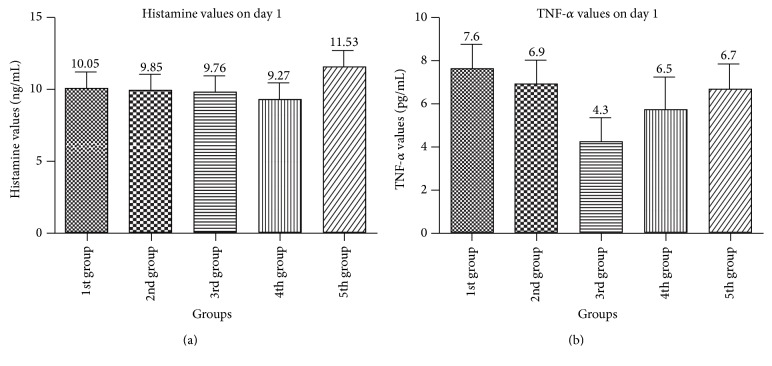
Comparison of first day histamine and TNF-*α* values between all groups.

There was no statistically significant difference between groups when we compared the first day histamine and TNF-*α* values (Figure 2).

Second (LPS) group histamine and TNF-*α* levels (mean ± SD) were 9.85 ± 3.58 ng/mL and 6.9 ± 0.9 pg/mL, respectively, at the beginning of the study. The levels rose to 34.14 ± 3.02 ng/mL and 119.2 ± 13.5 pg/mL at the end of the study. These changes were statistically significant (*P* < 0.001 for temporal changes of both) (Table 1).

Third (strontium) group histamine and TNF-*α* levels were 9.76 ± 3.61 ng/mL and 4.3 ± 2.1 pg/mL, respectively, at the beginning of the study. The levels rose to 11.33 ± 2.57 ng/mL and 27.8 ± 14.1 pg/mL at the end of the study. These changes were not statistically significant (*P*: 0.995 and 0.102) (Table 1).

Fourth (preventive) group end of study histamine level was 31.88 ± 2.58 ng/mL. Fifth (treatment) group end of study histamine level was 37.28 ± 4.16 ng/mL. When we compared the histamine levels of second (LPS) group (34.14 ± 3.02 ng) with those of fourth and fifth groups, the changes were not statistically significant (*P*: 0.942 and 0.702) (Table 2).

Fourth (preventive) group end of study TNF-*α* level was 28.0 ± 15.1 pg/mL. Fifth (treatment) group end of study TNF-*α* level was 25.0 ± 14.6 pg/mL. When we compared the TNF-*α* levels of second (LPS) group (119.2 ± 13.5 pg/mL) with those of fourth and fifth groups, the changes were statistically significant (*P* < 0.001 for both) (Table 3).

**Figure 3:**
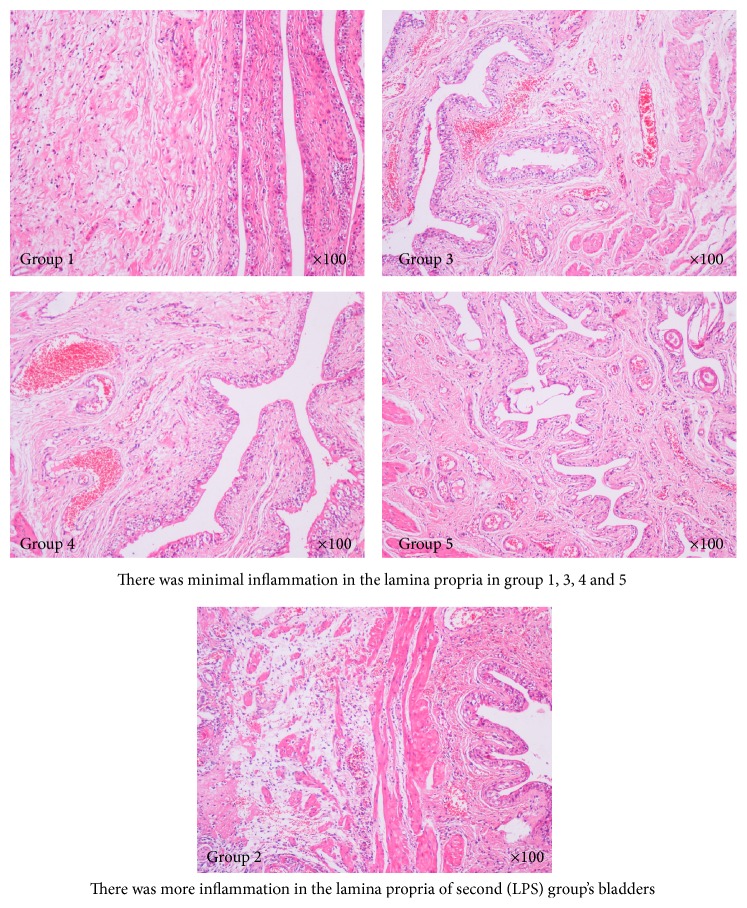
Histologic view of bladders with hematoxylin and eosin (H&E) staining protocol in all groups.

There was no significant difference between the groups histologically (Figure 3).

## 4. Discussion

IC/PBS is a chronic condition characterized by frequent and urgent urination, pain in the pelvis and/or incontinence, and variable inflammation. There are various animal models of IC but none of these models are not reliable. LPS has been used to induce experimental cystitis [11] like cyclophosphamide induced cystitis [12] or neurogenic cystitis [13]. In this study we used LPS to induce experimental cystitis model and we showed that SC significantly decreased urine TNF-*α* levels in treatment and preventive groups. To our knowledge this is the first study reporting that SC may decrease urine TNF-*α* in response to LPS. However, SC could not inhibit LPS induced urine histamine levels in both groups.

When SC was instilled to normal bladders, it did not make significant changes in bladder histology or urine histamine and TNF-*α* levels. We showed that SC significantly reduced the TNF-*α* levels of urine when applied as preventive and treatment purpose in experimental cystitis model induced by LPS. Interestingly, in the same model, strontium chloride did not affect urinary histamine levels.

Svensson et al. demonstrated that cartilage had a 60% lower rate of synthesis of glycosaminoglycans and collagen on the rats which received oral strontium therapy in their study [14]. Due to the negative impact of strontium on cartilage glycosaminoglycan levels, we thought it could adversely affect the bladder glycosaminoglycan layer. However, there were no significant changes in urinary histamine and TNF-*α* values or the bladder histopathology when we applied strontium to normal bladder intravesically. These findings suggested that strontium does not have any negative effect on bladder glycosaminoglycan layer in contrast to the effect on cartilage.

The primary role of TNF-*α* is in the regulation of immune cells and it can also induce apoptotic cell death through activation of the transcription factor, nuclear factor kappa-light-chain enhancer of activated B cells, and c-Jun N-terminal kinase [15]. Shie et al. showed that apoptosis of urothelial cells in patients with IC/PBS could result from upregulation of inflammatory signals, including p38 mitogen-activated protein kinase and TNF-*α* [5]. Another study's findings indicate that TNF-*α* mainly induces urothelium apoptosis via TRAIL pathway and TRAIL-R4 is the predominant receptor in the interstitial cystitis inflammation [6].

In experimental studies, urine and bladder tissue TNF-*α* levels were found to be increased when the cystitis was induced with LPS [16, 17]. In our study, in accordance with these publications, when bladders were instilled with LPS, TNF-*α* levels increased in the urine samples of rats.

Previous studies have shown that elevated levels of urinary histamine are commonly observed in patients with IC/PBS [18] and are often used as a diagnostic factor for this condition [19]. Therewithal previous experimental studies demonstrated that urinary histamine levels were increased in LPS treated animals [11, 16, 17]. Topical strontium, formulated in water-soluble salts, has been shown to be able to reduce histamine induced itch [20]. The exact mechanism underlying the antipruritic effect of strontium salts is not well understood. Although strontium reduced the histamine-induced itch, it did not change urine histamine levels significantly in the model of our study.

Topal et al. recently demonstrated that oral strontium chloride hexahydrate (40 mg/kg and 160 mg/kg) suppressed serum TNF-alpha levels and had comparable therapeutic efficiency when compared with prednisolone in a rat ulcerative colitis model [21]. Buache et al. demonstrated that the use of a strontium-substituted biomaterial can decrease the production of inflammatory cytokines including TNF-*α*, IL-6 and slowing down osteoclastogenesis [22]. In addition Fromigué et al. showed that strontium ranelate prevented cell apoptosis induced by serum deprivation or the proinflammatory cytokines, IL-1*β* and TNF-*α*, in regardless CaSR activation status, osteoblasts [23]. Furthermore in another study, Sr-Ca coadministration resulted in decreased expression of TNF-*α* in large animal model of osteoporosis [10].

Cytokine expressions, including lymphocytes and immune cells, depend on calcium influx through the cell membranes. Intracellular calcium increases providing calcium-calmodulin complex and calcineurin causes proinflammatory cytokine expression such as TNF-alpha and IL-1. Strontium competitively blocks the influx of calcium and may block proinflammatory cytokines expression indirectly [24, 25]. The relationship between strontium and TNF-*α* which is shown in these publications supports our finding in this study.

The lack of measurements of the amount of TNF and the expression of TNF-*α* gene in the bladder tissue is the major limitation of this study.

## 5. Conclusion

In our interstitial cystitis model, created with lipopolysaccharide, SC did not make any significant changes in histopathology of the bladders and the levels of histamine; however, it significantly reduced the levels of TNF-*α*. Given the role of TNF-*α* in the physiopathology of interstitial cystitis, these results suggested that SC may be a promising agent for the treatment of IC/PBS and further studies are required to evaluate the potential use of strontium in the management of interstitial cystitis.

## Figures and Tables

**Table 1:** The changes of histamine and TNF-*α* levels in second (LPS) and third (strontium) groups.

	Mean ± SD at the beginning (on day 1)	Mean ± SD at the end (on day 7)	*P*
Second group histamine	9.85 ± 3.58 ng/mL	34.14 ± 3.02 ng/mL	<0.001
Second group TNF-*α*	6.9 ± 0.9 pg/mL	119.2 ± 13.5 pg/mL	<0.001
Third group histamine	9.76 ± 3.61 ng/mL	11.33 ± 2.57 ng/mL	0.995
Third group TNF-*α*	4.3 ± 2.1 pg/mL	27.8 ± 14.1 pg/mL	0.102

**Table 2:** Comparison of end study histamine values of second (LPS) group with those of fourth (preventive) and fifth (treatment) groups.

Second (LPS) group histamine value on day 7 (34.14 ± 3.02 ng/mL)	Fourth (preventive) group histamine value on day 7	*P*
	31.88 ± 2.58 ng/mL	0.942
	Fifth (treatment) group histamine value on day 7	*P*
	37.28 ± 4.16 ng/mL	0.702

**Table 3:** Comparison of end study TNF-*α* values of second (LPS) group with those of fourth (preventive) and fifth (treatment) groups.

Second (LPS) group TNF-*α* value on day 7 (119.2 ± 13.5 pg/mL)	Fourth (preventive) group TNF-*α* value on day 7	*P*
	28.0 ± 15.1 pg/mL	<0.001
	Fifth (treatment) group TNF-*α* value on day 7	*P*
	25.0 ± 14.6 pg/mL	<0.001

## Abbreviations and Acronyms

IC/PBS: Interstitial cystitis/painful bladder syndrome
LPS: Lipopolysaccharide
TNF: Tumour necrosis factor
SC: Strontium chloride.
